# A Zero-Padding Frequency Domain Convolutional Neural Network for SSVEP Classification

**DOI:** 10.3389/fnhum.2022.815163

**Published:** 2022-03-17

**Authors:** Dongrui Gao, Wenyin Zheng, Manqing Wang, Lutao Wang, Yi Xiao, Yongqing Zhang

**Affiliations:** ^1^School of Computer Science, Chengdu University of Information Technology, Chengdu, China; ^2^School of Life Science and Technology, University of Electronic Science and Technology of China, Chengdu, China; ^3^National Key Laboratory of Human Factors Engineering, China Astronaut Research and Training Center, Beijing, China; ^4^School of Computer Science and Engineering, University of Electronic Science and Technology of China, Chengdu, China

**Keywords:** electroencephalogram, zero-padding frequency domain, steady-state visual evoked potential, steady-state motor visual evoked potential, convolutional neural network

## Abstract

The brain-computer interface (BCI) of steady-state visual evoked potential (SSVEP) is one of the fundamental ways of human-computer communication. The main challenge is that there may be a nonlinear relationship between different SSVEP in other states. For improving the performance of SSVEP BCI, a novel CNN algorithm model is proposed in this study. Based on the discrete Fourier transform to calculate the signal's power spectral density (PSD), we perform zero-padding in the signal's time domain to improve its performance on the PSD and make it more refined. In this way, the frequency point interval in the PSD of the SSVEP is consistent with the minimum gap between the stimulation frequency. Combining the nonlinear transformation capabilities of CNN in deep learning, a zero-padding frequency domain convolutional neural network (ZPFDCNN) model is proposed. Extensive experiments based on the SSVEP dataset validate the effectiveness of our method. The study verifies that the proposed ZPFDCNN method can improve the effectiveness of the SSVEP-based high-speed BCI ITR. It has massive potential in the application of BCI.

## 1. Introduction

Brain-Computer Interface (BCI) is a kind of communication system which converts the “ideas” in the brain into instructions. It can directly communicate with the machine to express intentions and ideas without language or actions. In the past few decades, among various modes of BCI, the BCI of SSVEP realized by EEG has been widely concerned and studied because of its high ITR, high signal-to-noise ratio, less training time, and reliability (Bin et al., 2009). It has been widely used in many fields such as medical diagnosis, rehabilitation for the disabled (Lebedev and Nicolelis, 2017), entertainment experience, and other fields, and it has made a considerable contribution to improving the quality of life of the disabled (Gao et al., 2003). Although BCI based on SSVEP has demonstrated high application value in various fields, its design and application still need to be studied, and it is still facing enormous challenges.

Traditional algorithms for detecting SSVEP signals can be roughly divided into four categories. (1) Spectrum analysis method based on Fourier transform, such as the fast Fourier transform (FFT) and power spectral density analysis (PSDA). (2) Methods based on signal decomposition analysis, such as the Hilbert-Huang transform (HHT) algorithm (Huang et al., 1998). (3) Algorithms based on canonical correlation analysis (Lin et al., 2006; Bin et al., 2009), such as multi-way canonical correlation analysis (MwayCCA) (Yu et al., 2011), filter bank canonical correlation analysis (FBCCA) algorithm (Chen et al., 2015), individual template-based canonical correlation analysis (IT-CCA) (Bin et al., 2011), L1 regularized multi-channel canonical correlation analysis (L1-MCCA) (Zhang et al., 2013). (4) Algorithms based on spatial filter and template matching, such as correlated component analysis (CORCA) (Zhang et al., 2018), task-related component analysis (TRCA) (Nakanishi et al., 2018), sum of squared correlations (SSCOR) (Kiran Kumar and Ramasubba Reddy, 2019), multi-stimulus task-related component analysis (msTRCA) (Chi et al., 2020). The first type of algorithm is relatively simple, has a short computation time, and is suitable for single channels. However, the calculation requires long enough signal data, and it is necessary to assume that the signal is linear and steady. This type of algorithm cannot handle highly complex EEG signals with nonlinear and non-stationary features (Cheng et al., 2002; Wang et al., 2006; Chen et al., 2017). The second type of algorithm analyzes the signal in the time-frequency domain; it has better versatility in processing nonlinear and non-stationary signals than FFT. However, in the face of highly complex SSVEP signals, its performance is still unsatisfactory (Huang et al., 2008). The third algorithm detects the SSVEP signal by calculating the correlation between the EEG and reference signals. But, this kind of algorithm cannot well deal with nonlinear relations in real signals (Bin et al., 2009), and there is a certain gap in accuracy and information transmission rate compared with methods in supervised training (Nakanishi et al., 2018). Although the fourth type of algorithm is a supervised training method dependent on subjects, it cannot extract specific subject and task-related information from individual calibration data in many application scenarios of SSVEP-based BCI. It is not conducive to the application and popularization of SSVEP-based BCI. At the same time, visual fatigue, inattention, and other factors that are not independent of individual subjects will also affect the performance of the algorithm independent of the subjects.

Recently, deep learning has been successfully applied in many fields and has achieved remarkable results in the classification task of SSVEP signals. Zhang et al. proposed an external convolutional neural network (CNN) to detect the intentional control (IC) state and unintentional control (NC) state in EEG. The results clearly show that the proposed shallow CNN method can distinguish between IC and NC states in EEG (Zhang et al., 2019b). In addition, the steady-state motion visual evoked potential (SSMVEP) BCI system detects multiple sub-states in the IC state. Some researchers have proposed a novel convolutional neural network (FFT-CNN-CCA) to see the NC state and multiple IC sub-states in the SSMVEP-BCI system (Zhang et al., 2019a). Gao et al. introduced the deep learning (DL) method in a cart control system designed based on SSMVEP signals. The results show that the constructed deep learning model of a convolutional neural network with long and short-term memory (CNN-LSTM) is not only suitable for “EEG illiterate” people but can significantly improve the performance of “EEG illiterate” people (Gao et al., 2020). The deep learning method has been widely used in EEG, EMG, and other signals (Waytowich et al., 2018; Ravi et al., 2020; Zhang et al., 2021). Nevertheless, in the classification task of SSVEP signals with a large number of categories, few articles mention that the performance of the deep learning algorithm model exceeds some existing spatial filter algorithms, such as the TRCA (Nakanishi et al., 2018) or msTRCA (Chi et al., 2020) algorithm. The performance and ITR of SSVEP-based BCI applications largely depend on the classification accuracy of SSVEP signals under more stimulus targets and shorter time windows. However, these deep learning methods have not been studied on many stimulus targets or validated on standard public datasets. Moreover, how to improve the classification accuracy of SSVEP's BCI through deep learning methods is still to be studied.

Therefore, this article proposes a zero-padding frequency domain convolutional neural network (ZPFDCNN) model to solve the above problems. Because the spectrum calculated by discrete Fourier transform (DFT) has Picket Fence Effect, padding zeros at the end of the signal can increase the point density and reduce the sampling error of the spectrum calculated by DFT. At the same time, the zero-padding method does not introduce any frequency component into the intercepted signal but can improve the observed value of the movement in the spectrum. So, inspired by it, we use the zero-padding method to calculate the PSD of the signal and then combined with the nonlinear transformation ability of CNN, the ZPFDCNN method is proposed. This study aims to use the deep learning method to classify SSVEP signals and SSMVEP signals with multiple stimulation target frequencies to prove the effectiveness of the ZPFDCNN method based on deep learning training SSVEP-BCI.

The main contributions of this article are as follows: (1) In the feature extraction of SSVEP and SSMVEP signals caused by cycle visual stimuli, a feature extraction method is proposed for calculating the PSD of a signal with zero-padding in the time domain. This method can effectively extract frequency information in the SSVEP and SSMVEP signals. (2) Considering the impact of visual delay on classification accuracy, studying different harmonic numbers on the impact of the proposed classification model, by selecting the corresponding harmonic number and fusion multi-channel information, the different categories SSVEP and SSMVEP signals of nonlinear transform capability are combined. (3) On the public BETA: SSVEP dataset and the SSMVEP training dataset of the 2020 World Robot Contest-BCI Controlled Robot Contest, offline experiment results show that the method is better than existing TRCA and msTRCA methods.

## 2. Method

### 2.1. Method Overview

**Figure 1:**
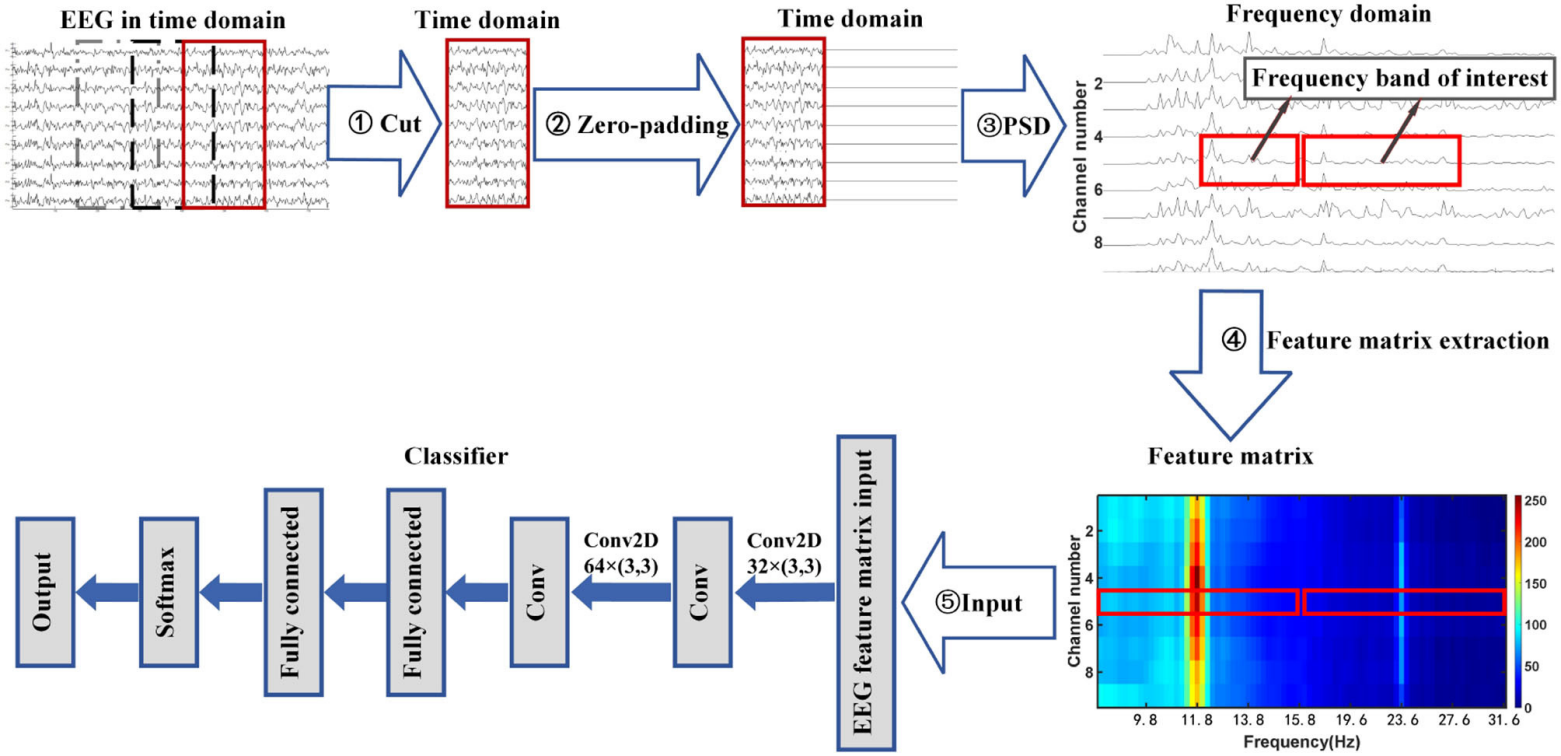
Flowchart of zero-padding frequency domain convolutional neural network algorithm for SSVEP recognition.

This article proposes a zero-padding frequency domain convolutional neural network model method to identify different types of SSVEP signals or SSMVEP signals. The structure flow chart of this method is shown in Figure 1. It can be seen from the figure that the process consists of three parts. First, The EEG signal is intercepted and padded with zeros in the time domain to improve the observed value of the signal's PSD in the frequency domain. Then, the fundamental frequency band and the second harmonic frequency band from the PSD of the nine-channel SSVEP signal or SSMVEP signal are extracted and combined into a feature matrix, which retains useful information while removing unnecessary information interference. Finally, the feature matrix is used as the input of the algorithm model to identify different types of SSVEP signals or SSMVEP signals by nonlinear transformation.

### 2.2. Introduction to the Dataset

#### 2.2.1. Introduction to the BETA:SSVEP Dataset

The BETA:SSVEP dataset (Liu et al., 2020) comes from 70 healthy subjects, including 28 women, 42 men, age range 9–64 years, average age: 25.14 years, standard deviation: 7.97 years. The user interface of the BCI speller in the experiment corresponding to this dataset is a 5*8 stimulus matrix containing 40 characters. Use linearly increasing frequency and phase to encode 40 characters. The frequency range is 8–15.8 Hz with 0.2 Hz intervals. The phase value starts from 0, and the interval is 0.5π . The dataset has been bandpass filtered between 3 and 100 Hz to remove ambient noise and then epoch extraction. Starting from each block, they include 0.5 s before stimulation, 2 s of stimulation (for S1–S15) or 3 s of stimulation (for S16–S70), and 0.5 s after stimulation. After that, the data of all periods are downsampled to 250 Hz.

#### 2.2.2. Introduction to SSMVEP Dataset

The SSMVEP dataset comes from the SSVEP training dataset of the 2020 World Robot Contest-BCI Controlled Robot Contest with a sampling frequency of 1,000 Hz, including a reference dataset and a training dataset. The training dataset includes the A list dataset and the B list dataset. The reference dataset contains a total of 20 subjects' experimental data. A total of three EEG data collections were performed for each subject. Each EEG data collection contains 35 stimulation targets. Both the A list training dataset and the B list training dataset include the experimental data of six subjects; Each subject has collected the EEG data twice. Each EEG data collection is also a stimulus containing 35 stimulus targets. The stimulus target appears randomly in each experiment, and each stimulus target appears once.

The experimental paradigm of the SSMVEP dataset uses a circular checkerboard of periodic radial contraction-expansion movement as the paradigm of visual stimulation. The stimulation paradigm of this dataset contains a total of 35 stimulation targets. The stimulation frequency of the stimulation targets is 3–20 Hz with an interval of 0.5 Hz. The initial phase of each stimulation target is 0.5π. The experimental data in the SSMVEP dataset is based on a block, and each block contains continuously collected EEG data. The individual trials in the experimental data lasted 5 s, including 3 s visual stimulation phase and 2 s rest phase. In the process of experimental visual stimulation, 35 targets were presented simultaneously. The presentation of each stimulus target changes sinusoidally according to its preset frequency. Subjects were asked to watch the prompted target strictly to evoke a steady-state visual evoked potential in their brain electrical signals.

### 2.3. EEG Signal Preprocessing

#### 2.3.1. BETA:SSVEP Dataset Preprocessing

In the BETA: SSVEP dataset, Liu et al. (2020) have organized each subject's data into a separate mat file. So we do not need to start with the original EEG signals. Each subject's mat file contains a four-dimensional double type matrix with a variable name of EEG and a structure called suppl_info that includes the subject's practical information. The size of the matrix is 64*750/1000*4*40. Each dimension represents the number of channel indexes, the number of data points, the number of blocks, and the number of stimulation sequences. The suppl_info structure contains some practical information about the subjects.

In the process of evaluating the algorithm model, EEG data was selected from nine electrodes (Pz, PO5, PO3, POz, PO4, PO6, O1, Oz, O2), and it is filtered out unnecessary noise in the filter function by a 5–100 Hz IIR bandpass filter designed in MATLAB. Considering the influence of visual latency mentioned by Liu et al. (2020), a latency of 130 ms was applied to suppress the effect of visual latency on model classification.

#### 2.3.2. SSMVEP Dataset Preprocessing

##### 2.3.2.1. Filtering

The data of the SSMVEP dataset is the original EEG signal data without any processing. Each subject is stored in a mat file, consisting of a two-dimensional array. The two dimensions of the array represent the number of channels and the number of sampling points, respectively. Among them, the last channel saves the tags and synchronously records the event information in the experiment; It contains the label at the start time and the end time, the label at the start time and the end time of the stimulus.

In the process of evaluating the model, EEG data was selected from nine electrodes (Pz, PO5, PO3, POz, PO4, PO6, O1, Oz, O2), and it is filtered out unnecessary noise in the filter function by a 2–102 Hz IIR bandpass filter designed in MATLAB.

##### 2.3.2.2. Data Collation

The filtered SSMVEP data is trimmed and sorted by retrieving the 65th channel label. Each subject's data is saved as a four-dimensional matrix. Each matrix dimension represents the number of channel indexes, the number of data points collected, the number of blocks, the number of stimuli ordinal. Among them, the collected data points include 1000 sampling points one second before the start of stimulation, in visual stimulation, and one second after the end of visual stimulation. Organize the data in this way to facilitate post-processing.

##### 2.3.2.3. Consideration of Visual Latency

**Figure 2:**
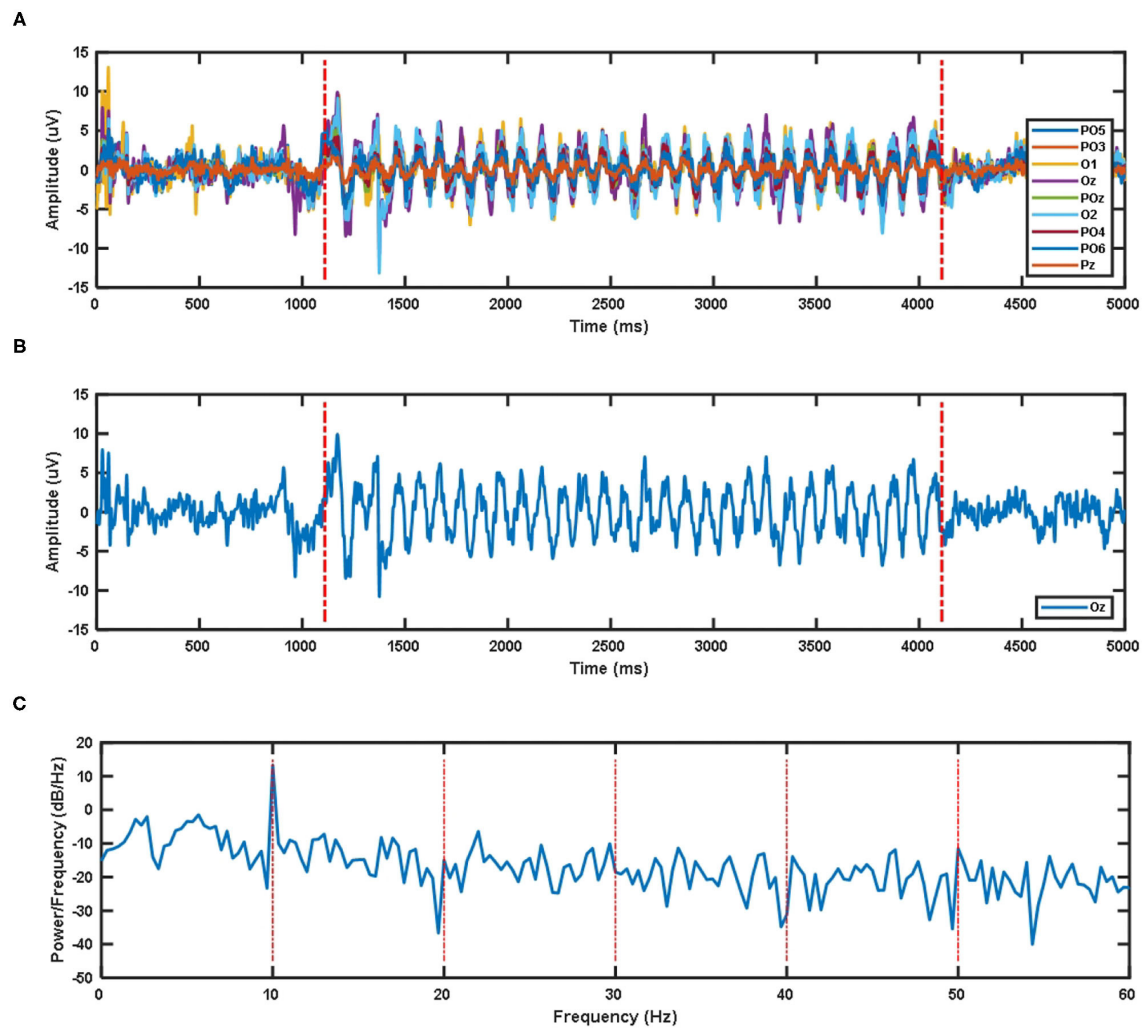
**(A)** The superposition average of time domain waveforms of nine electrode channels at 10 Hz visual stimulation frequency for all subjects. **(B)** Superposition average of time domain waveform of Oz electrode channel at 10 Hz visual stimulation frequency for all subjects. **(C)** Superposition average of PSD of Oz electrode channel at 10 Hz visual stimulation frequency for all subjects.

Due to the impact of visual latency on the classification of EEG signals, we have considered the visual latency of the EEG data in the SSMVEP dataset. First, all EEG data was filtered and processed before. Then the EEG signals of all subjects under nine electrodes (Pz, PO5, PO3, POz, PO4, PO6, O1, Oz, O2) and 35 different stimulation frequencies were superimposed and averaged. After sorting, the average time-domain waveforms of nine electrode channels of all subjects under 10 Hz visual stimulation frequency are shown in Figure 2A. The two red dashed lines are the artificially estimated start time of the stimulation target and the end time of the stimulation target.

The time-domain waveform and PSD of the Oz electrode channel EEG signal at 10 Hz visual stimulation frequency are shown in Figure 2B. The two red dotted lines in the time-domain waveform are the artificially estimated stimulus target start time and the stimulus target end time. Figure 2C's PSD shows that the SSMVEP signal has a very significant amplitude performance at the fundamental frequency of the stimulation target, and it has almost no corresponding amplitude response at the multiple of the stimulation frequency. Therefore, this may be why the stimulus paradigm for data collection in this dataset does not consider the influence of the doubling frequency of the stimulus frequency.

**Figure 3:**
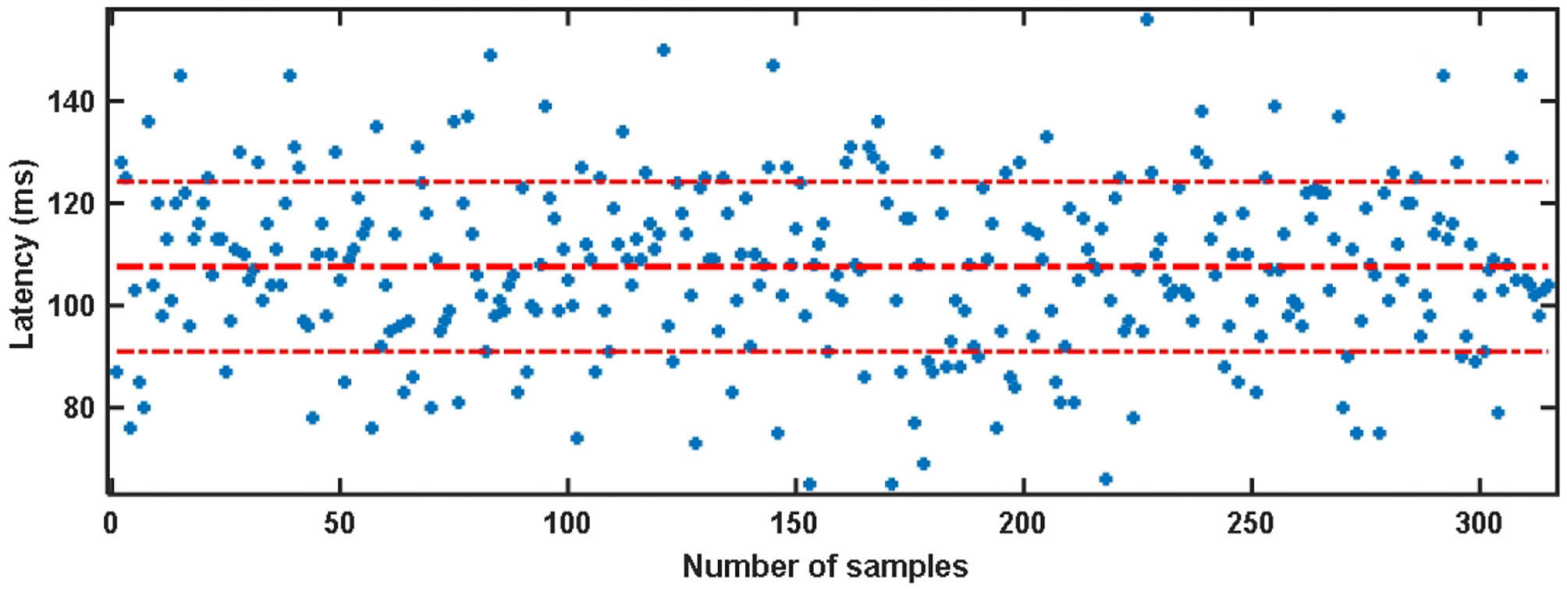
Artificial estimation of the visual latency and its average value in the SSMVEP dataset.

The visual latency of all subjects in the SSMVEP dataset was estimated manually. The method is to superimpose and average the EEG data of 35 stimulation frequencies under the nine electrodes (Pz, PO5, PO3, POz, PO4, PO6, O1, Oz, O2) of each subject. Then we analyzed and estimated the visual latency in the EEG collected by them and estimated the average visual latency and standard deviation in the system as shown in Figure 3. The average value of the artificially estimated visual latency is 107.61 ms, and the standard deviation is 16.63 ms.

### 2.4. Time Domain Zero-Padding Feature Extraction

#### 2.4.1. Features of SSVEP and SSMVEP Signals

Both SSVEP signal and SSMVEP signal are weak evoked EEG signals. It is susceptible to the influence of other brain electricity and noise interference. At the same time, factors such as the participant's status and the participant's attention strategy will also affect the detection of the SSVEP signal. This makes the SSVEP signal have a big difference between different subjects. Age and gender have a certain degree of influence on SSVEP. Therefore, it is difficult to distinguish other SSVEP signals purely from the perspective of time-domain waveforms.

Because both SSVEP signal and SSMVEP signal are EEG signals induced by periodic visual stimulation, they have apparent characteristics in the frequency domain. For the SSVEP, one of the most prominent features is relatively strong amplitude performance at the fundamental frequency point of the corresponding stimulation frequency and the harmonic frequency point of the doubling frequency in the frequency domain. The SSMVEP signal has a less harmonic amplitude than the SSVEP signal. It can hardly see the second harmonic, and it cannot see the higher-order harmonics at all (Han et al., 2018). One of its most prominent features is its relatively strong amplitude performance at the fundamental frequency point corresponding to the stimulation frequency on the frequency domain spectrum.

Therefore, the signal detection difficulties for SSVEP and SSMVEP mainly have the following three aspects. (1) The spectrum diagram in the frequency domain makes it easy to distinguish different SSVEP signals or SSMVEP signals. However, in frequency domain spectrum analysis, the signal needs to reach a certain data length which makes it easy to distinguish SSVEP signals or SSMVEP signals with different stimulation frequencies (Cheng et al., 2002; Wang et al., 2006; Chen et al., 2017). (2) The induced SSVEP signal and SSMVEP signal frequency bands are relatively wide. But, the amplitude of EEG induced by different stimulation frequencies is different. Moreover, SSVEP will be affected by harmonics. This leads to the fact that the stimulation frequency band and the corresponding response frequency band used in the BCI are relatively narrow. (3) To increase the ITR of BCI, more visual stimulation frequencies are selected in the SSVEP signal or the SSMVEP signal with a narrower stimulation response frequency band. This makes the frequency interval between the different SSVEP and SSMVEP signals smaller, making it more difficult to distinguish different SSVEP signals or SSMVEP signals in the frequency domain.

#### 2.4.2. Improvement of PSD by Zero-Padding

The frequency resolution of the frequency spectrum in the discrete Fourier transform (DFT) can be understood as the minimum frequency interval that can be obtained on the frequency axis when using the discrete Fourier transform.


(1) $$\begin{array}{l} f_{0}=\frac{F_{s}}{N}=\frac{1}{Nt_{s}}=\frac{1}{T} \end{array}$$

where *N* is the number of sampling points, *F*_*s*_ is the sampling rate. *t*_*s*_ is the sampling interval. So *Nt*_*s*_ is the time length *T* of the analog signal before sampling. Therefore, the longer the signal length, the better the signal spectrum frequency resolution. The Fourier Transform (FT) is a linear integral transform used to transform the signal in the time domain to the frequency domain. The continuous Fourier transform *X*_*f*_ is defined as


(2) $$\begin{array}{l} X\left(f\right)=\int_{-\infty }^{\infty }x\left(t\right){\text{e}}^{-\text{j}2\pi f\text{t}}dt \end{array}$$

where *x*(*t*) is a continuous signal in the time domain. *X*(*f*) is the continuous spectrum of the signal in the frequency domain, *t* represents the time axis of the signal, and *f* represents the frequency axis of the signal. DFT is a discrete form of Fourier Transform. The DFT in the frequency domain of the discrete sequence *X*(*m*) is defined as:


(3) $$\begin{array}{l} X\left(m\right)=\overset{N-1}{\underset{n=0}{\sum }}x\left(n\right){\text{e}}^{-j2\pi nm/N} \end{array}$$

this is the DFT equation in exponential form. Among them, *x*(*n*) is the discrete sampling value of the time-domain continuous variable *x*(*t*), *n* is the discrete sampling of *t*. “e” is the base of the natural logarithm, and the imaginary number symbol j= $\sqrt{-1})$. *n* is the discrete sampling point on the frequency axis of the signal's bilateral spectrum behind the discrete Fourier transform. The value range is the same as *m*, ranging from 0, 1, 2, 3, to N-1.

The DFT of a sequence of *N* points can only observe the spectrum on a limited number of *N* frequency points. It is equivalent to observing the scenery from the gap of the fence. Sometimes it is not enough to understand the features of EEG signals in the entire frequency domain. To observe the information on other frequency points, it is necessary to process the original signal *x*(*n*) to get more samples on the frequency points. Increase the original number of sampling points in the DTFT frequency domain to *M* the point, so that the sampling point position becomes


(4) $$\begin{array}{l} {\left\{\omega_{k}^{\prime }=e^{ik\frac{2\pi }{M}}\right\}}_{0\leq k<M} \end{array}$$

then the corresponding DFT becomes


(5) $$\begin{array}{l} {\hat{x}}^{\prime }\left[k\right]=\hat{x}\left(e^{ik\omega_{k}^{\prime }}\right)=\overset{N-1}{\underset{n=0}{\sum }}x\left[n\right]e^{-i\frac{2\pi }{M}kn} \end{array}$$

where *k* represents the discrete sampling point of *M* discrete sampling points on the frequency axis of the discrete Fourier transform bilateral spectrum, with a value range of 0,1,2, …, M-1. If *M*−*N* zeros are added after the sequence *x*[*n*] and set as x′[n], the above formula becomes


(6) $$\begin{array}{l} {\hat{x}}^{\prime }\left[k\right]=\overset{M-1}{\underset{n=0}{\sum }}x^{\prime }\left[n\right]e^{-i\frac{2\pi }{M}kn}=Fx^{\prime } \end{array}$$

Therefore, the value *x*[*n*] 's DTFT at other frequency points can be obtained by adding zero to *x*[*n*] 's and then doing DFT. This is equivalent to moving the fence to be observed at other frequency points.

The above conclusion can be verified by the theory of finite DFT. Let the EEG signal x(nΔ) = [x(0), x(Δ), …, x(N−1)Δ]. The sampling interval of the EEG signal is Δ. Then after Fourier transform, the frequency spectrum of *x*[*nΔ*] is


(7) $$\begin{array}{l} \begin{array}{c} x\left(nd\right)=\overset{N-1}{\underset{n=0}{\sum }}x\left(n\Delta \right)\cdot \exp\left(-i2\pi \;mn/N\right)\;\left(m=0,1,\cdots \;,N-1\right) \end{array} \end{array}$$

among them


(8) $$\begin{array}{l} d=1/N\Delta \end{array}$$

*d* is the resolution when the length of the EEG signal is *N* .

If we add *M* zeros to the EEG signal *x*[*nΔ*], that is to say


(9) $$\begin{array}{l} x^{\prime }\left(n\Delta \right)=\left(x\left(0\right),x\left(\Delta \right),\cdots \;,x\left(N-1\right)\Delta ,0,\cdots \;,0\right) \end{array}$$

then the spectrum of *x*′(*nΔ*) after transformation is


(10) $$\begin{array}{l} \begin{array}{c} x^{\prime }\left(md1\right)=\overset{M+N-1}{\underset{n=0}{\sum }}x^{\prime }\left(n\Delta \right)\cdot \exp\left(-i2\pi \;mn/\left(N+M\right)\right)\\ =\overset{N-1}{\underset{n=0}{\sum }}x\left(n\Delta \right)\cdot \exp\left(-i2\pi \;mn/\left(M+N\right)\right)\\ |\left(m=0,1,\cdots \;,M+N-1\right) \end{array} \end{array}$$

where


(11) $$\begin{array}{l} d1=1/\left(M+N\right)\Delta \end{array}$$

*d*1 is the resolution of the EEG signal when padding *M* zeros. It can be seen from Equations (7) and (10) that for the same value of *m*


(12) $$\begin{array}{l} x\left(md\right)\neq x^{\prime }\left(md1\right) \end{array}$$

but at the same frequency point, that is


(13) $$\begin{array}{l} md=m1d1 \end{array}$$

then there is


(14) $$\begin{array}{l} m_{1}=md/d1=m\left(M+N\right)/N \end{array}$$

thus


(15) $$\begin{array}{l} \begin{array}{c} x^{\prime }\left(m1d1\right)=\overset{M+N-1}{\underset{n=0}{\sum }}x^{\prime }\left(n\Delta \right)\cdot \exp\left(-i2\pi m1n/\left(M+N\right)\right)\\ =\overset{N-1}{\underset{n=0}{\sum }}x\left(n\Delta \right)\exp\left(-i2\pi mn/N\right)=x\left(md\right) \end{array} \end{array}$$

The above formula derivation proves that the spectrum observed using the zero-padding technique and not the zero-padding approach is consistent. However, the zero-padding can reduce the interval between the various frequency points on the frequency domain spectrum after the DFT of the signal. It can reduce the influence of the “Picket Fence Effect” caused by continuous Fourier transform to DFT in the frequency domain and improve the observation of signals in the frequency domain. At the same time, due to the nature of the DFT, an input signal whose frequency component in the intercepted signal is not at an integer multiple of the minimum frequency interval of the DFT frequency domain. It will leak to other DFT output frequency units. For a cosine wave with k cycles on the N-point input time series, the frequency unit amplitude response of the N-point DFT (the frequency unit index is represented by m) is approximately equal to the sinc function. For a cosine wave with k cycles on the N-point input time series, the frequency unit amplitude response of the N-point DFT (the frequency unit index is represented by m) is approximately equal to the sinc function.


(16) $$\begin{array}{l} X\left(m\right)=\frac{N}{2}\cdot \frac{\sin\left[\pi \left(k-m\right)\right]}{\pi \left(k-m\right)} \end{array}$$

this formula can determine the magnitude of the signal leakage on the spectrum after the DFT. The truncation of the signal in the time domain is equivalent to multiplying the signal by a rectangular window in the time domain. The multiplication of signals in the time domain is equivalent to convolution in the frequency domain. Therefore, the DFT of the signal will convolve a sinc function on each frequency component in the frequency domain. So, an appropriate frequency interval in the frequency domain can reduce the impact caused by spectrum leakage to a certain extent. At the same time, because the rectangular window has the smallest main lobe width on the DFT unit, it is easier to obtain a clear and distinguishable signal spectrum than others, such as Hamming windows and triangular windows. The feature extraction part of the ZPFDCNN algorithm model we proposed is to extract the PSD features of the EEG signal in the frequency domain. The PSD spectrum is calculated based on the Fourier transform. The calculation of the PSD under the continuous Fourier transform is as follows.


(17) $$\begin{array}{l} P\left(f\right)=\underset{T\to \infty }{\lim}\frac{|X\left(f\right){|}^{2}}{2\pi T} \end{array}$$

among them, *P*(*f*) is the PSD of the signal, and *X*(*f*) is the frequency spectrum after the DFT of the signal. The calculation of the PSD in the discrete case is shown in the following formula.


(18) $$P(m)=\frac{{\left|X(m))\right|}^{2}}{F_{s}*N}$$

among them, *P*(*m*) is the PSD when the signal is discrete. *X*(*m*) is the frequency spectrum under the DFT of the signal. *F*_*s*_ is the sampling rate of the signal under discrete conditions. *N* is the number of sampling points of the signal under discrete conditions.

#### 2.4.3. The Steps of Feature Extraction

**Figure 4:**
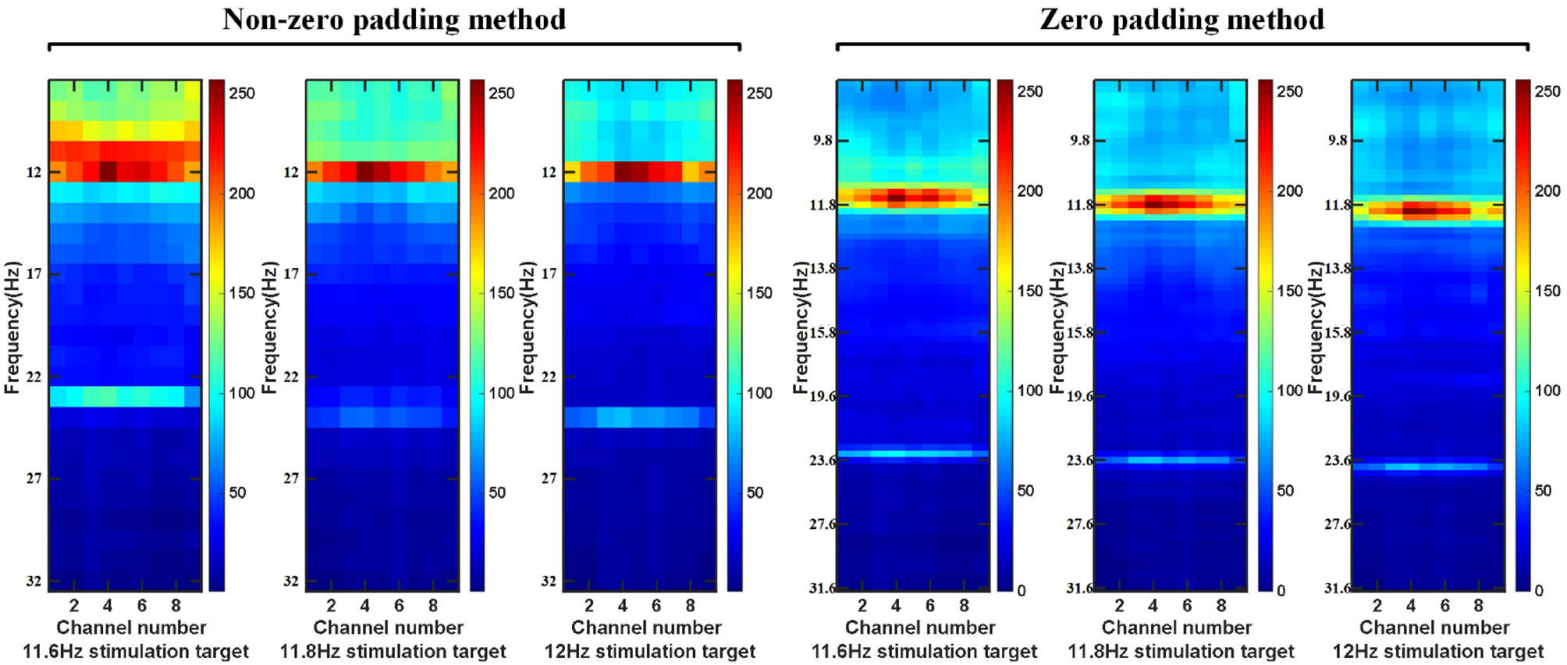
In BETA:SSVEP dataset, feature matrix after superposition and average of PSD without zero-padding and PSD with zero-padding.

First of all, for the preprocessed EEG data, we use a sliding window to obtain a single EEG data sample on the epoch between the stimulation starts with the visual latency and the stimulation ends with the visual latency. The step size of the sliding window is a data length of 0.1 s. The overlap time of the data is the size of the sliding window minus the sliding step length. Then, the intercepted signal is zero-padded to make the frequency point interval of the signal consistent with the minimum frequency interval between the stimulation frequency. Perform feature extraction on the data of nine electrodes (Pz, PO5, PO3, POz, PO4, PO6, O1, Oz, O2) channels in the BETA: SSVEP dataset and SSMVEP dataset. Finally, considering the influence of the harmonic sub-band on the model classification, the 80*9 dimension feature matrix comprises the fundamental frequency band amplitude data and the second harmonic frequency band amplitude data. In the BETA: SSVEP dataset, under the 1.0 s time window of the highest ITR of the ZPFDCNN algorithm model, the superimposed average visualization of feature matrices of 11.6, 11.8, and 12 Hz categories are shown in Figure 4. Compared with the PSD estimation without zero-padding, the PSD estimation with zero-padding technology further expands the difference between different categories. It makes it easier to distinguish different types of SSVEP signals.

Using this feature extraction has the following six advantages. (1) This technology reduces the interval between the frequency points of the spectrum and improves the resolution between the frequency points of the signal spectrum. (2) In DFT, the rectangular window function with the minimum main lobe weakens the influence between adjacent frequency points compared with other window functions. (3) The normalization processing after feature extraction makes the data distribution more reasonable. (4) Compared with the direct use of PSD, the zero-padding method of feature extraction increases the “spacing” between various categories, which is more conducive to the classification of the deep learning model. (5) The frequency feature is one of the most prominent features of SSVEP and SSMVEP. DFT can transform signals from aliasing frequency information in the time domain to different dimensions in the frequency domain, which is equivalent to a part of feature extraction. (6) For the two datasets, using the frequency band information of interest in the frequency domain removes some noise interference. It reduces the input dimension, model complexity, and training time compared with the time domain information directly as the input.

### 2.5. Frequency Domain Convolution Classifier

#### 2.5.1. Convolutional Network Structure

This research designed the convolutional neural network according to the extracted signal features as shown in the following Table 1. The neural network consists of five consecutive layers: two convolutional layers, two fully connected layers, and an output layer.

**Table 1:** The structure of the CNN model used in the BETA:SSVEP dataset and SSMVEP dataset.

**Number**	**Type**	**Description**
1	Matrix input	80*9*1 Matrices with “rescale-zero-one” normalization
2	Convolution	32 3*3 convolutions with stride [1 1] and padding “same”
3	Batch normalization	Batch normalization
4	Leaky ReLU	Leaky ReLU with scale 0.01
5	Convolution	64 3*3 convolutions with stride [1 1] and padding “same”
6	Batch normalization	Batch normalization
7	Leaky ReLU	Leaky ReLU with scale 0.01
8	Fully connected	2048 fully connected layer
9	Batch normalization	Batch normalization
10	Leaky ReLU	Leaky ReLU with scale 0.01
11	Fully connected	Fully connected layer(BETA:SSVEP dataset:40 neurons; SSMVEP dataset:35 neurons)
12	Batch normalization	Batch normalization
13	Leaky ReLU	Leaky ReLU with scale 0.01
14	Softmax	Softmax
15	Classification output	Cross entropyex

The input data has been preprocessed and feature extracted as described above. Then, the harmonic characteristics of SSVEP are considered simultaneously in the ZPFDCNN method. The data of fundamental frequency band (BETA: SSVEP dataset: 8–15.8 Hz, SSMVEP dataset: 3–20 Hz) and harmonic frequency band (BETA: SSVEP dataset: 16–31.6 Hz, SSMVEP dataset: 6–40 Hz) are used as the input of the network. Layer 1 and Layer 2 are two-dimensional convolution layers. In Layer 1 and 2, 32 and 64, 3*3 convolution kernels are used for convolution, respectively. The 3*3 convolution kernel has been proven to perform well in the image field. The article shows that in the case of the same receptive field, multiple 3*3 convolution kernels have more nonlinear functions than a more significant size convolution kernel, which increases the nonlinear expression and makes the classification decision function more difficult deterministic. At the same time, in the case of having the same receptive field, the former has fewer parameters, which reduces the amount of calculation in the convolution kernel. It is more conducive to increasing the model's depth or accelerating its training speed. Layer 3 is fully-connected in the CNN model with 2,048 neurons. Layer 4 is also fully connected. However, the number of classification categories determines the number of neurons in this layer. In the BETA: SSVEP dataset, the number of neurons in Layer 4 is 40. In the SSMVEP dataset, the number of neurons in Layer 4 is 35. Batch normalization makes the data output distribution of each layer more reasonable. It can accelerate the training speed and increase the model's generalization ability. The Leaky ReLU activation function can increase the model's nonlinear transformation capability. Therefore, the batch normalization and Leaky ReLU activation functions are used for all previous layers. Layer 5 is the output layer, using the softmax function. The classification loss function is mutually exclusive classes' cross-entropy of Kc (Kind of category).

### 2.6. Training Parameters

The learning of network weights uses the Adam optimization algorithm that combines the momentum gradient and RMSprop algorithms. The optimization algorithm can further reduce the jitter of the update and balance the update speed of each parameter, speed up the convergence, and ensure the convergence. The algorithm is computationally efficient and used with very few memory requirements. It is very suitable for more significant problems in terms of data and parameters. The algorithm is ideal for non-stationary targets and issues with very noisy and sparse gradients. The algorithm uses error backpropagation to optimize network weights, and the loss function uses a cross-entropy function. The learning rate is set to 0.0001. The model assesses the number of training epochs to 30, and the batch size in stochastic gradient descent is 512.

## 3. Result

### 3.1. Evaluating Indicator

#### 3.1.1. Information Transmission Rate

Information translate rate (ITR) was originally used for the communication and calculation rate of measurement systems in the communication field. It was introduced into the BCI field by Wolpaw and is an important indicator for measuring the performance of BCIs in the BCI field. The calculation formula is as follows:


(19) $$\begin{array}{l} ITR=\frac{60}{T}\left[\log_{2}Q+P\log_{2}P+\left(1-P\right)\log_{2}\frac{1-P}{Q-1}\right] \end{array}$$

among them, *T* represents the average trial duration, which includes the duration of the time window and the duration of the attention shift. *Q* represents the number of targets, and P represents the recognition accuracy rate. The unit of *ITR* is bits/min. For calculating the theoretical ITR for offline analysis, a gaze shift time of 0.55 s is chosen according to the previous studies (Chen et al., 2015; Wang et al., 2017), which was proven sufficient in an online spelling task (Chen et al., 2015).

#### 3.1.2. Classifier Performance Quantification

Accuracy (*ACC* ) and confusion matrix can be used to measure the algorithm's performance in the BETA:SSVEP dataset and SSMVEP dataset. The calculations were carried out in two datasets, respectively. Among them, *FPR*, *TPR*, *ACC* can be easily calculated through the confusion matrix. The definitions of the three indicators are as follows:


(20) $$\begin{array}{l} FPR=\frac{FP}{FP+TN} \end{array}$$

(21) $$\begin{array}{l} TPR=\frac{TP}{TP+FN} \end{array}$$

(22) $$\begin{array}{l} ACC=\frac{TP+TN}{TP+FN+FP+TN} \end{array}$$

among them, *FP*, *TN*, *TP*, *FN* are the number of false positives, true negatives, true positives, and false negatives, respectively. In our research, positive refers to the state of correct classification, and negative refers to the state of incorrect classification. the 10-fold cross-validation was performed on the two datasets. Both datasets are divided into 10 sub-samples of equal size. Among the 10 sub-samples, one sub-sample is reserved as verification data to test the model, and the remaining nine sub-samples are used as training data. There is no overlap between the training subset and the test subset. The cross-validation process was repeated ten times, and each sample data in the ten sub-sample data was verified once.

### 3.2. Performance

#### 3.2.1. Performance in the BETA: SSVEP Dataset

**Figure 5:**
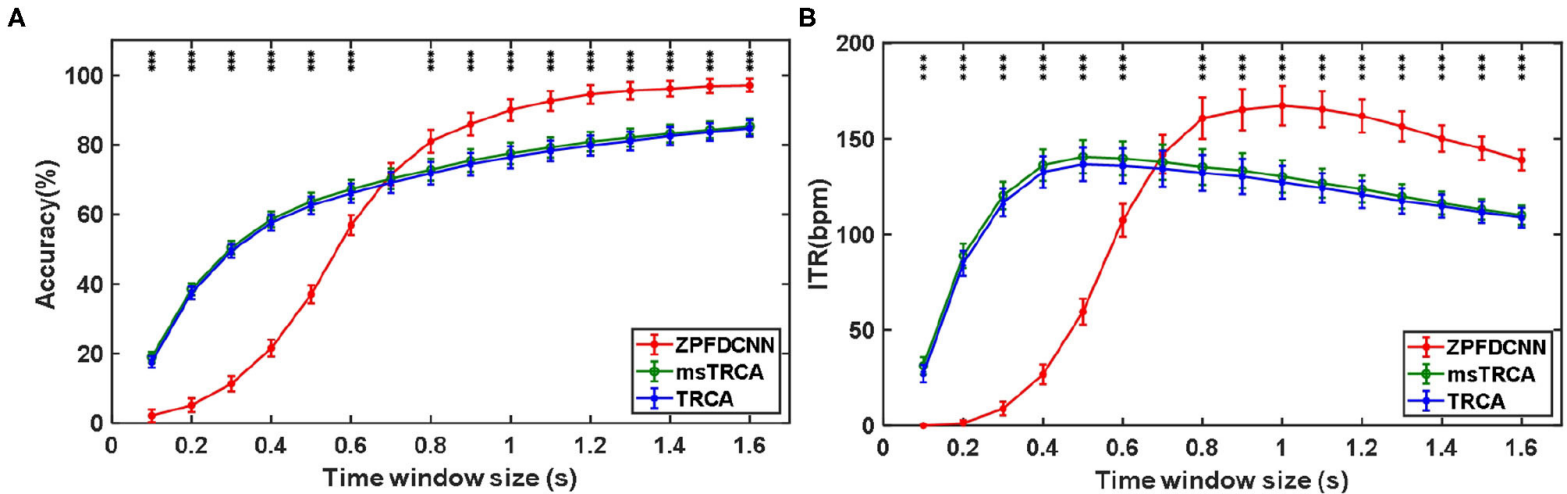
**(A)** Average classification accuracy of the BETA:SSVEP dataset. **(B)** The average ITR of the BETA:SSVEP dataset with a visual shift time of 0.55 s. The error bars indicate standard errors. Note that * denotes *p* < 0.05, ** denotes *p* < 0.01, *** denotes *p* < 0.001 according to one-way repeated measure ANOVAs.

**Figure 6:**
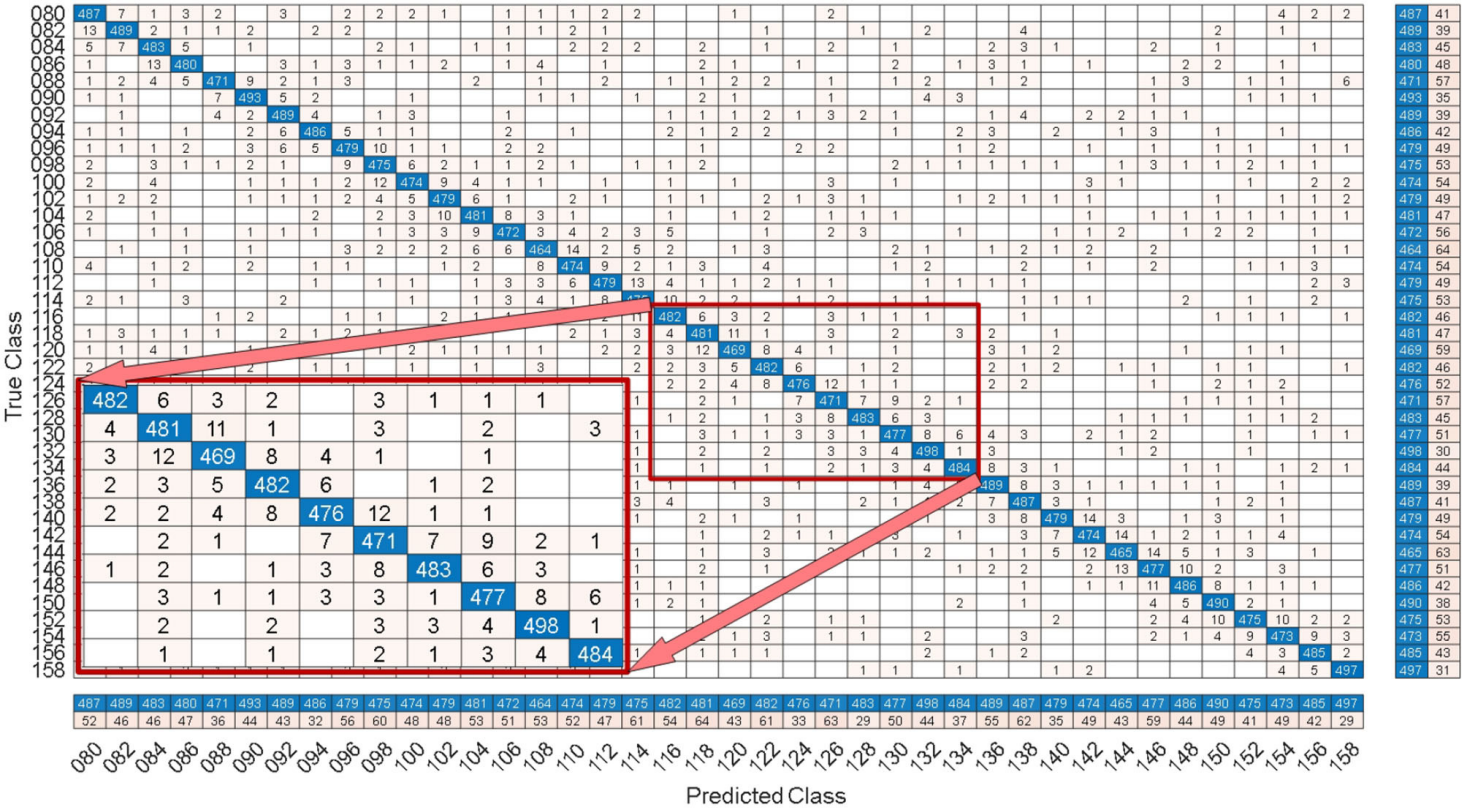
Under the time window of 1.0 s, the confusion matrix for one of the 10-fold cross-validation of the BETA:SSVEP dataset.

By comparing with methods based on TRCA (Nakanishi et al., 2018) and msTRCA (Chi et al., 2020), we studied the performance of our proposed ZPFDCNN algorithm model on the BETA: SSVEP dataset. The filter bank technology can significantly improve the classification accuracy based on TRCA and msTRCA methods. Therefore, we explored algorithms based on TRCA and msTRCA under five sub-bands. For simplicity, we will refer to them as the TRCA algorithm and the msTRCA algorithm in the following. Figure 5 compares the classification accuracy percentage and ITR for all subjects at different time windows. In this figure, one-way repeated measure ANOVAs were performed to test whether there was a significant difference between the three methods. The comparison of the Figure 5 and Table 2 shows that the classification accuracy and ITR of the proposed ZPFDCNN model are significantly better than the other two methods from 0.7 s and after the time window. In the 1.0 s time window, the average classification accuracy rate is 89.99%, reaching the highest ITR: 167.36 bit/min. The ZPFDCNN method is significantly better than the msTRCA method, which is achieved 63.75% classification accuracy and ITR: 140.65 bit/min under a time window of 0.5 s. In Figure 6, the confusion matrix diagram is one of the ten-fold crossvalidation of the BETA:SSVEP dataset under a 1.0 s time window. It can see from Figure 6 that the ZPFDCNN algorithm model can effectively distinguish the SSVEP signals between different stimulation frequencies through the confusion matrix diagram. Meanwhile, it also has enough classification ability to distinguish two adjacent stimulus frequency points. Under the time window >0.7 s, the ZPFDCNN method is superior to TRCA and msTRCA. The possible reasons are as follows: (1) In the frequency domain, the feature extraction method of the zero-padding calculation PSD can be extracted to more spectrum information than direct calculation PSD. (2) Compared with the algorithm based on correlation analysis, the nonlinear transformation ability of the deep learning algorithm is more reliable in classification. In the time window of fewer than 0.7 s, the ZPFDCNN algorithm performs weaker than TRCA and msTRCA. The possible reason is that the PSD information calculated by the interception signal under the shorter time window is insufficient to support the model to distinguish between different categories. At this time, the performance of the ZPFDCNN is not comparable to the algorithm based on correlation analysis.

**Table 2:** Average classification accuracy and ITR of the BETA:SSVEP dataset with a visual shift time of 0.55 s.

**Time window size(ms)**	**TRCA (%)**	**msTRCA (%)**	**ZPFDCNN (%)**	**TRCA (bpm)**	**msTRCA (bpm)**	**ZPFDCNN (bpm)**
100	17.59	19.01	2.13	27.25	31.36	0.03
200	37.42	38.52	5.16	84.84	88.87	1.29
300	49.51	50.49	11.35	116.72	120.38	8.90
400	57.65	58.63	21.56	132.65	136.21	26.788
500	62.61	63.75	37.03	**136.69**	**140.65**	59.589
600	66.09	67.26	56.91	135.93	139.77	107.39
700	69.15	70.26	71.56	134.40	137.84	141.95
800	71.82	72.85	81.02	132.22	135.26	160.79
900	74.42	75.50	85.94	130.33	133.40	165.23
1,000	76.45	77.55	89.99	127.33	130.33	**167.36**
1,100	78.26	79.25	92.61	124.27	126.84	165.49
1,200	79.78	80.90	94.51	120.9	123.73	162.00
1,300	81.10	82.10	95.57	117.52	119.93	156.52
1,400	82.53	83.23	96.12	114.79	116.40	150.17
1,500	83.65	84.29	96.87	111.67	113.11	145.05
1,600	84.75	85.32	97.09	108.83	110.08	138.94

*The optimal ITR performance of each algorithm is marked in bold*.

#### 3.2.2. Performance in the SSMVEP Dataset

**Figure 7:**
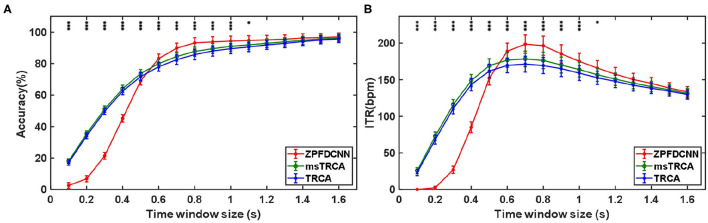
**(A)** Average classification accuracy of the SSMVEP dataset. **(B)** The average ITR of the SSMVEP dataset with a visual shift time of 0.55 s. The error bars indicate standard errors. Note that * denotes *p* < 0.05, ** denotes *p* < 0.01, *** denotes *p* < 0.001 according to one-way repeated measure ANOVAs.

**Figure 8:**
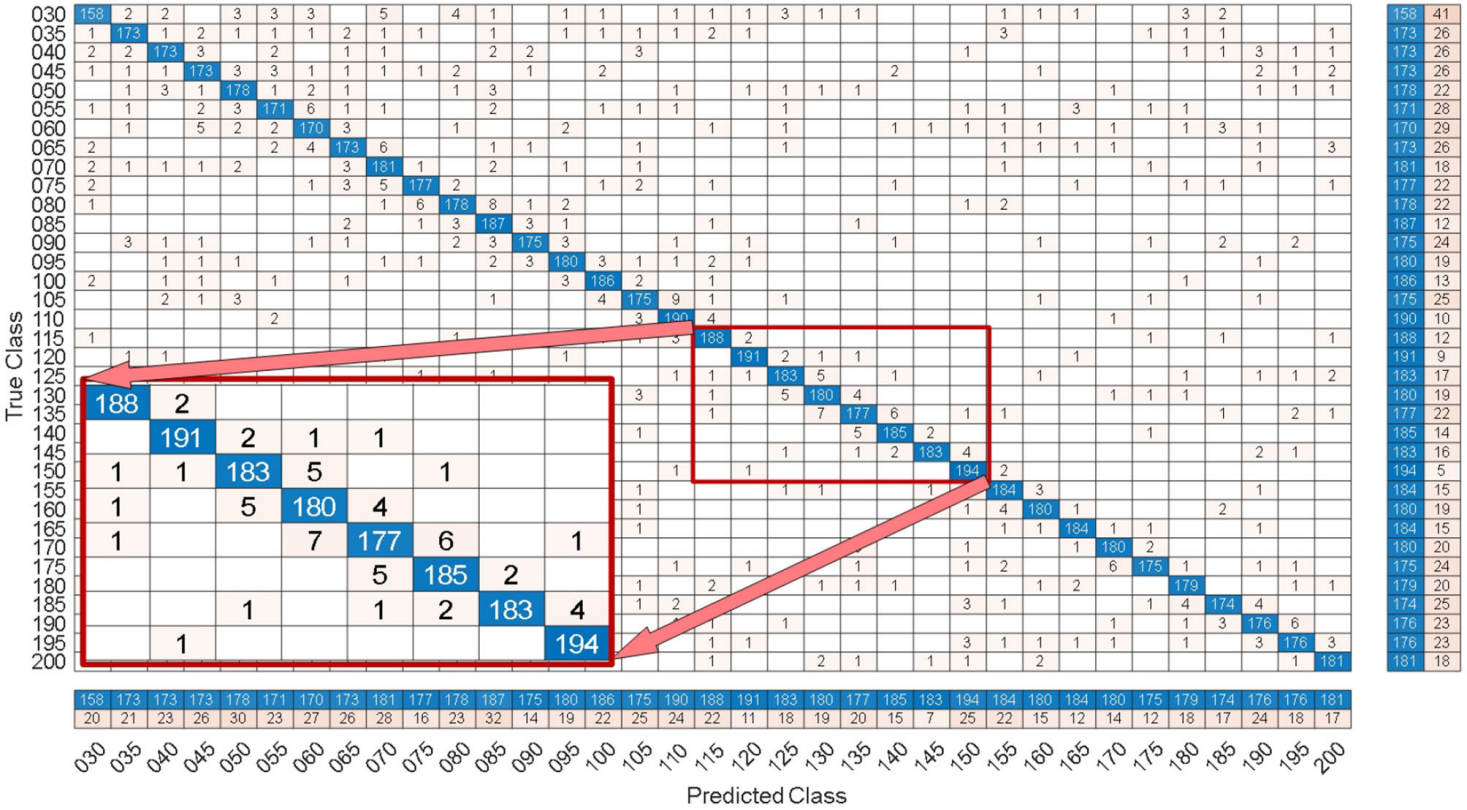
Under the 0.7 s time window, the confusion matrix of one of the 10-fold cross validation of the SSMVEP dataset.

By comparing with methods based on TRCA (Nakanishi et al., 2018) and msTRCA (Chi et al., 2020), we studied the performance of our proposed ZPFDCNN algorithm model on the SSMVEP dataset. As mentioned above, the filter bank technology (five sub-bands) is used to improve the classification accuracy based on the TRCA and msTRCA methods. Figure 7 shows the comparison of classification accuracy and ITR for all subjects in different time windows. In this figure, one-way repeated measure ANOVAs were performed to test whether there was a significant difference between the three methods. From comparing of the Figure 7 and Table 3, the ZPFDCNN algorithm model we proposed is significantly better than the other two methods in classification accuracy and ITR starting from 0.6 s time window and after. Moreover, in the time window of 0.7 s, the average classification accuracy rate is 89.84%, reaching the highest ITR: 198.64 bit/min. The ZPFDCNN algorithm model is significantly better than the msTRCA method, which reaches 84.50% classification accuracy and the highest ITR: 178.49 bit/min in the time window of 0.7 s. At the same time, the confusion matrix in Figure 8, one of the 10-fold cross-validation, also shows the excellent classification performance of the ZPFDCNN algorithm model. Compared with the BETA: SSVEP dataset, the ZPFDCNN method's performance is better in the SSMVEP dataset. The possible reasons are as follows: (1) Fewer classification categories. (2) Compared with the stimulation frequency interval of 0.2 Hz and the frequency bandwidth of 8–15.8 Hz in the BETA: SSVEP dataset, the stimulation frequency interval of the SSMVEP dataset is more extensive, reaching 0.5 Hz, and the frequency band to evoke visual stimulation is wider: 3–20 Hz. The latter contains more helpful information at the same sampling rate and time length, which is more conducive to classifying different categories. To make the frequency interval of the calculated PSD spectrum consistent with the interval between stimulation frequencies, the feature extraction method in this dataset will use fewer zeros for padding.

**Table 3:** Average classification accuracy and ITR of SSMVEP dataset with a visual shift time of 0.55 s.

**Time window size(ms)**	**TRCA (%)**	**msTRCA (%)**	**ZPFDCNN (%)**	**TRCA (bpm)**	**msTRCA (bpm)**	**ZPFDCNN (bpm)**
100	17.05	18.01	2.51	23.11	25.65	0.03
200	34.10	35.52	6.81	68.07	72.83	2.36
300	49.61	51.10	21.37	110.54	115.91	26.86
400	62.53	64.02	45.16	143.30	148.80	85.02
500	71.73	73.72	69.08	161.83	169.23	152.23
600	78.01	80.00	83.21	169.60	176.87	188.99
700	82.51	84.50	89.84	**171.40**	**178.49**	**198.64**
800	85.72	87.73	93.16	169.40	176.37	196.51
900	88.01	89.51	93.81	165.11	170.12	185.36
1,000	89.49	90.99	94.31	159.08	163.91	175.15
1,100	90.56	91.90	94.73	152.65	156.81	165.94
1,200	91.88	92.89	95.07	147.78	150.78	157.54
1,300	92.84	93.84	95.48	142.48	145.37	150.28
1,400	94.02	94.80	96.32	138.41	140.61	145.07
1,500	95.01	95.70	96.53	134.33	136.23	138.59
1,600	95.67	96.27	97.03	129.81	131.43	133.54

*The optimal ITR performance of each algorithm is marked in bold*.

#### 3.2.3. Ablation Experiment

**Figure 9:**
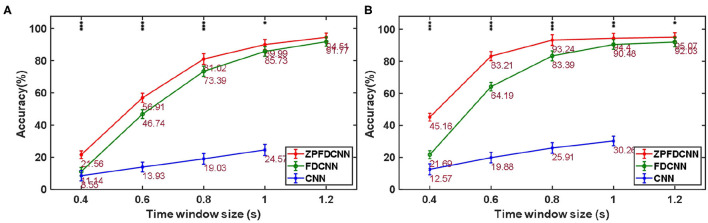
Comparison among the ZPFDCNN method, the FDCNN method, and the CNN method. **(A)** Comparison on BETA:SSVEP datset. **(B)** Comparison on SSMVEP dataset. The error bars indicate standard errors. Note that * denotes *p* < 0.05, ** denotes *p* < 0.01, *** denotes *p* < 0.001 according to one-way repeated measure ANOVAs.

To compare the influence PSD method with zero-padding and the PSD method without zero-padding on CNN model classification. We only change the size of the CNN model input when other conditions remain unchanged. Figure 9 shows the comparison between calculating the SSVEP's PSD with zero-padding and convolution (the ZPFDCNN method), calculating the SSVEP's PSD without zero-padding and convolution (the FDCNN method), and the method of directly using the original signal as the network input (the CNN method) and due to the high sampling rate of SSMVEP dataset, directly using the original EEG signal as input is easy to make GTX 2080TI burst video memory. Therefore, the data is downsampled to 250 Hz. In the CNN method with the original data as the input, the data dimension of 1.2 s is too high, which leads to the explosion of video memory. So, in the 1.2 s data length, neither BETA: SSVEP dataset nor the SSMVEP dataset can get the result of the CNN method classification. It can be seen from the figure that the performance of the ZPFDCNN method is better than the FDCNN method and CNN method. One-way repeated measure ANOVAs are performed to test whether there was a significant difference between the ZPFDCNN method and the FDCNN method. The results of 10-fold cross-validation are shown in Figure 9. Meanwhile, we compared the training time of the ZPFDCNN method, the FDCNN method, and the CNN method when other training parameters remained unchanged. The comparison results are shown in Table 4. Compared with the CNN method, the training time of the ZPFDCNN method is also reduced due to the reduction of input dimension.

**Table 4:** Comparison of training once time of the ZPFDCNN method, the FDCNN method, and the CNN method in BETA:SSVEP dataset (under the time window of 1.0 s) and SSMVEP dataset (under the time window of 0.7 s).

**Training time (minutes)**	**ZPFDCNN**	**FDCNN**	**CNN**
BETA:SSVEP dataset	51	14	198
SSMVEP dataset	12	4	26

#### 3.2.4. The Influence of Separable Convolutions on ZPFDCNN Model

Separable convolutions have been widely used in the field of deep learning (Zhang et al., 2017; Zhang R. et al., 2019; Huang et al., 2020). It divides a kernel into two smaller kernels, were most common is to divide a 3*3 kernel into a 3*1 and 1*3 kernel. Hence, instead of conducting one convolution with nine multiplications, two convolutions with three multiplications each are done. Therefore, based on the ZPFDCNN method, the most common separable convolutions are applied to two convolution layers, respectively (This method is hereinafter referred to as the S-ZPFDCNN method). The 10-fold cross-validation results of the two ways are shown in Table 5. As can be seen from Table 5, the ability of S-ZPFDCNN to classify SSVEP and SSMVEP is not as good as ZPFDCNN. The possible reason is that not all kernels can be divided into two smaller ones.

**Table 5:** The comparison of the average accuracy of the ZPFDCNN method and the S-ZPFDCNN method after 10-fold cross-validation in BETA:SSVEP dataset and SSMVEP dataset.

**BETA:SSVEP dataset**			**Acc(%)**		
	Time(s)	0.6	0.8	1.0	1.2
	ZPFDCNN	56.91	81.02	89.99	94.51
	S-ZPFDCNN	35.17	56.71	71.09	83.17
**SSMVEP dataset**			**Acc(%)**		
	Time(s)	0.5	0.7	0.9	1.1
	ZPFDCNN	69.08	89.84	93.81	94.73
	S-ZPFDCNN	41.65	63.79	75.99	84.31

#### 3.2.5. The Influence of Different Harmonic Sub-band Numbers on Classification

**Figure 10:**
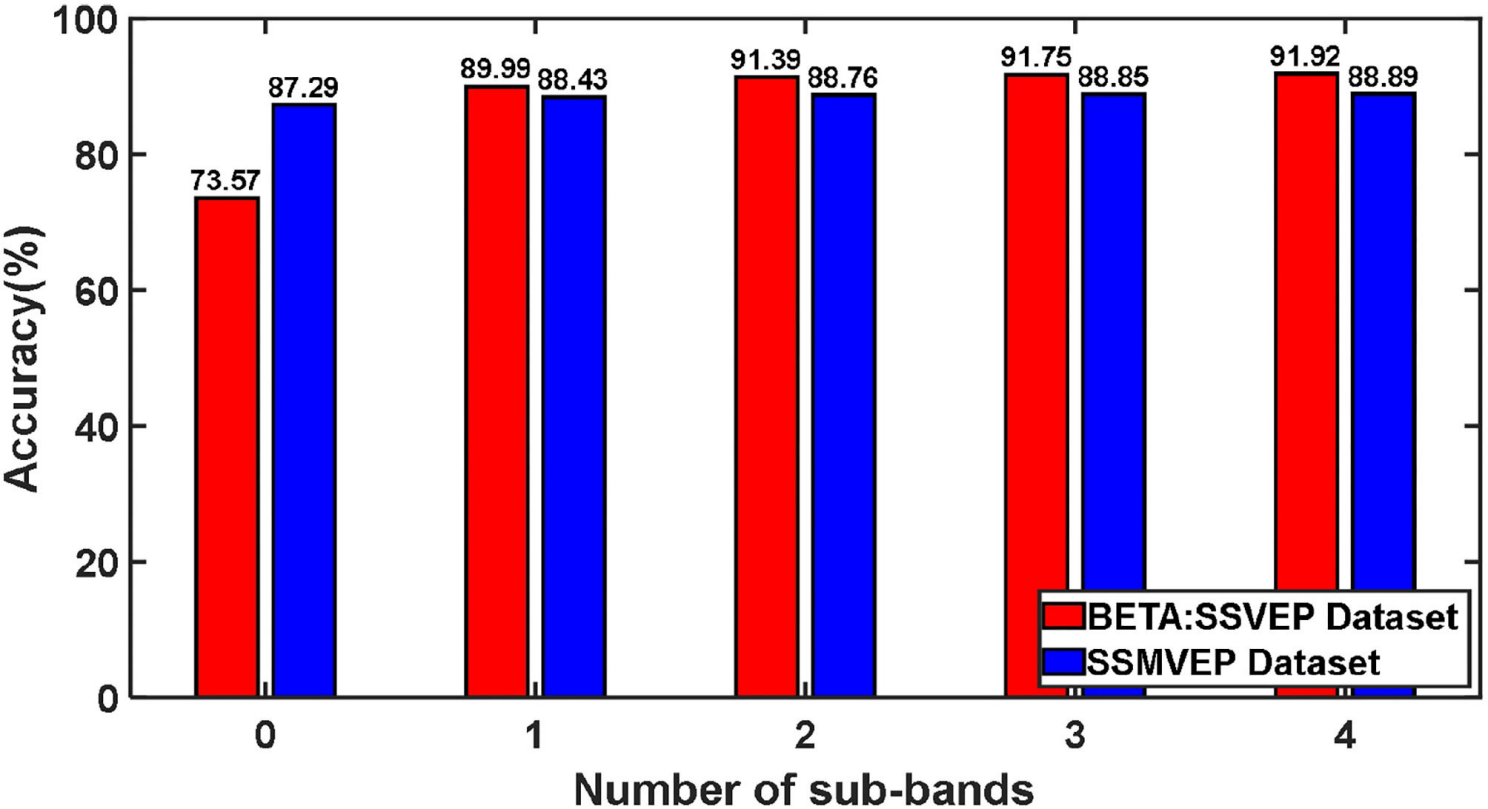
The influence of different harmonic frequency band numbers on the ZPFDCNN method.

We tested the influence of different harmonic sub-band numbers in the ZPFDCNN algorithm model's frequency identification of SSVEP and SSMVEP signals. We only change the size of the feature matrix input of the ZPFDCNN algorithm model when other parameters remain unchanged. Furthermore, the test was carried out under the time window of the optimal ITR performance of the BETA: SSVEP and SSMVEP datasets, respectively. Like the filter bank technology (Chen et al., 2015), when the feature matrix contains different numbers of harmonic sub-band, we verify its impact on the classification accuracy of the ZPFDCNN algorithm model. The average classification accuracy of the ZPFDCNN algorithm with ten-fold cross-validation under different harmonic sub-band numbers is shown in Figure 10.

In BETA: SSVEP dataset, the SSVEP signal is the EEG caused by the flashing stimulation of the black and white square flipping, which is a traditional classic SSVEP stimulation paradigm. This stimulation paradigm allows the brain to produce electrophysiological signals with the same frequency and multiples of frequency as visual stimulation. This also enables the harmonic components in the EEG signal to be used for signal classification and detection. From the above Figure 10, for traditional SSVEP signals, with the increase of the number of harmonic sub-band, the classification accuracy rate has been dramatically improved under the ZPFDCNN algorithm model. The improvement is significant, especially when harmonics are between 0 and 1. In the ZPFDCNN algorithm model, the harmonic frequency band of the traditional SSVEP signal stimulation frequency significantly impacts signal classification. In the SSMVEP dataset, we consider the visual latency of the SSMVEP signal. Then, we analyzed the PSD of all the subjects' Oz electrode channel EEG signals superimposed and averaged at a visual stimulation frequency of 10 Hz. The PSD shows that the SSMVEP signal has a very significant amplitude performance at the fundamental frequency of the stimulation target. But, there is almost no corresponding amplitude response at the octave frequency of the stimulation frequency. It can also be seen from the Figure 10, under the ZPFDCNN algorithm model, the number of harmonic frequency bands of the SSMVEP signal caused by the periodic radial contraction-expansion checkerboard stimulus paradigm has minimal effect on the classification accuracy. It is far less than the influence of harmonics in SSVEP induced by the traditional stimulation paradigm.

## 4. Discussion and Conclusion

The ZPFDCNN method has the following three advantages. (1) Compared with the direct calculation of PSD, the zero-padding method reduces the frequency point interval in the PSD calculated by the SSVEP signal. It reduces the sampling error caused by the “Picket Fence Effect” of DFT. It makes the PSD of SSVEP more accurate and improves the observation in the PSD of SSVEP. The comparison between the ZPFDCNN method and the FDCNN method also shows the effectiveness of the zero-padding method. (2) The traditional deep learning model takes the time-domain information of EEG data as the input; the ZPFDCNN method proposed in this study uses a whole frequency band. The harmonic band as input reduces the input dimension and reduces the complexity and training time of the model. In addition, it also reduces the impact of some noise. (3) Based on the improvement of spectrum by zero-padding method, the nonlinear ability of CNN is used to convolute in multi-channel and multi-band. The excellent classification performance of SSVEP signal and SSMVEP signal with many categories is realized.

In this study, we conclude that the CNN-based frequency domain convolutional neural network: the ZPFDCNN method. It is suitable for classifying SSVEP signals and SSMVEP signals with many categories. It can effectively improve the ITR of SSVEP-based high-speed BCI. The ZPFDCNN method based on CNN has excellent potential in various communication and control applications in the high-speed BCI of SSVEP.

## Data Availability Statement

The original contributions presented in the study are included in the article/supplementary material, further inquiries can be directed to the corresponding author/s.

## Author Contributions

YZ and YX supervised the study and designed the experiment. DG, WZ, MW, and LW perform the experiments and analysis. DG, WZ, and YZ wrote the manuscript. All authors have read and approved the final manuscript.

## Funding

This work was supported by the National Key Laboratory of Human Factor Engineering (No. SYFD061902K) and National Natural Science Foundation of China under Grant (No. 72071185).

## Conflict of Interest

The authors declare that the research was conducted in the absence of any commercial or financial relationships that could be construed as a potential conflict of interest.

## Publisher's Note

All claims expressed in this article are solely those of the authors and do not necessarily represent those of their affiliated organizations, or those of the publisher, the editors and the reviewers. Any product that may be evaluated in this article, or claim that may be made by its manufacturer, is not guaranteed or endorsed by the publisher.
